# Recent advances in structural studies of the CRISPR-Cas-mediated genome editing tools

**DOI:** 10.1093/nsr/nwy150

**Published:** 2018-11-29

**Authors:** Yuwei Zhu, Zhiwei Huang

**Affiliations:** HIT Center for Life Sciences, School of Life Science and Technology, Harbin Institute of Technology, Harbin 150080, China

**Keywords:** CRISPR-Cas system, adaptive immune, viral infection, genome editing

## Abstract

Clustered regularly interspaced short palindromic repeats (CRISPR) and accompanying CRISPR-associated (Cas) proteins provide RNA-guided adaptive immunity for prokaryotes to defend themselves against viruses. The CRISPR-Cas systems have attracted much attention in recent years for their power in aiding the development of genome editing tools. Based on the composition of the CRISPR RNA-effector complex, the CRISPR-Cas systems can be divided into two classes and six types. In this review, we summarize recent advances in the structural biology of the CRISPR-Cas-mediated genome editing tools, which helps us to understand the mechanism of how the guide RNAs assemble with diverse Cas proteins to cleave target nucleic acids.

## INTRODUCTION

**Figure 1.**
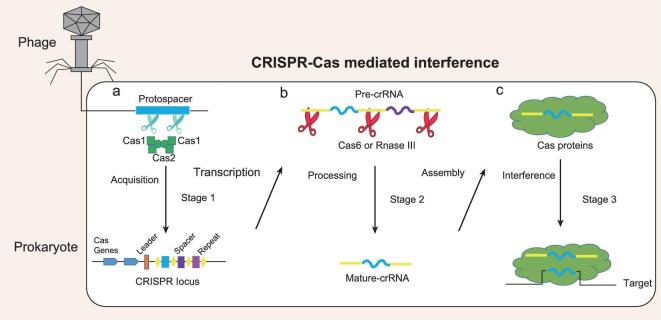
A cartoon depicting the general organization of a CRISPR-Cas locus and the three stages of CRISPR-Cas activity. (a) In the stage of spacer sequence acquisition, the Cas1 and Cas2 heterocomplex recognizes invading DNA (protospacer) and inserts it into the CRISPR array as a spacer sequence via a cut-and-paste mechanism. (b) In the stage of RNA (crRNA) biogenesis, a Cas6 or RNase III family nuclease processes the long transcript (pre-crRNA) from the CRISPR locus to a mature crRNA. (c) In the stage of RNA-guided interference, mature crRNAs associate with Cas proteins to form surveillance complexes, which recognize and cleave invading nucleic acids.

An evolutionary struggle between prokaryotes and viruses has been going on for billions of years [1]. The selective pressures imposed by viruses drive the diversification of the immune defense systems of prokaryotes [2,3]. Clustered regularly interspaced short palindromic repeats (CRISPR) and accompanying CRISPR-associated (Cas) proteins constitute an RNA-based antiviral immune system, found in about 90% of archaea and 50% of bacteria [4]. A typical CRISPR locus consists of an array of short direct repeats and interspersed spacer sequences, which is flanked by diverse *cas* genes [5] (Fig. 1a). The repeats contain the same sequences within a CRISPR locus, but vary in both length and sequence in different units. In contrast, the spacers present unique DNA sequences gained from invading viruses or plasmids. Adjacent to the first repeat of a CRISPR locus, an A–T-rich ‘leader’ sequence is observed, which is vital for CRISPR transcription and spacer acquisition [6,7] (Fig. 1a). The CRISPR-Cas adaptive immune systems are known to function through three distinct stages: spacer sequence acquisition (stage 1), CRISPR RNA (crRNA) biogenesis (stage 2) and RNA-guided interference (stage 3) [8,9]. During infection, a short sequence (protospacer) from an invading virus or plasmid is inserted into the CRISPR locus as a spacer [10,11] (Fig. 1a). Biochemical and structural biology studies have shown that Cas1 and Cas2 form a stable complex, serving as a governor for the incorporation of new spacers into the CRISPR locus via a cut-and-paste mechanism [12,13]. This acquisition machinery works in a sequence-specific manner to avoid self-targeting, so that only the invading DNA flanked by a protospacer-adjacent motif (PAM) can be recognized and selected as a protospacer. In the crRNA biogenesis stage, the CRISPR locus is transcribed into a precursor crRNA (pre-crRNA), which is subsequently processed into mature crRNAs (Fig. 1b). Pre-crRNA cleavage is mediated by either Cas6 (class 1 CRISPR-Cas systems) or RNase III (class 2 CRISPR-Cas systems) [14,15]. Finally, crRNA-guided interference occurs. In this stage, mature crRNAs associate with Cas proteins to form a surveillance complex, which recognizes and cleaves invading nucleic acids [16] (Fig. 1c).

Based on locus organization and gene conservation, the CRISPR-Cas systems can be divided into two classes, six types and several subtypes [17,18]. Class I CRISPR-Cas systems, consisting of types I, III and IV, employ multisubunit crRNA–effector complexes for interference. Class II CRISPR-Cas systems, consisting of types II, V and VI, feature the presence of a single subunit of a crRNA–effector module. The type I system is defined by the signature *cas3* gene, and is currently divided into seven subtypes (I-A, I-B, I-C, I-D, I-E, I-F and I-U) [18]. During the interference stage, multiple Cas proteins assemble with a mature crRNA to form the Cascade complex, which recruits a nuclease–helicase protein Cas3 to degrade invading nucleic acids [19]. Unlike the type I system, the type II CRISPR locus displays a simplified composition, utilizing a single effector protein (Cas9) guided by a dual-RNA heteroduplex (crRNA–tracrRNA (*trans*-activating crRNA)) [20,21]. The type II system can be further divided into three subtypes (II-A, II-B and II-C) [18]. The type III system employs a multiprotein complex, which is similar to that of the type I system. The signature gene of the type III system is *cas10*, which encodes a large multidomain protein. Four subtypes of the type III system have been identified to date, including III-A, III-B, III-C and III-D [18]. The type III system possesses two kinds of enzymatic activities (ssRNase and ssDNase) [22,23]. This property confers the type III system with a versatile immune response against different types of foreign genetic elements, and an efficient fail-safe way of degrading both the invading DNA and its transcript [24]. The type IV system is putative and functionally uncharacterized, showing a minimal multisubunit crRNA–effector complex that differs from the type I and type III systems [17]. *Csf1* is a hallmark gene of this system. The type V and VI systems utilize a single subunit crRNA–effector complex. Three RuvC domain-containing proteins (Cpf1, C2c1 and C2c3) have currently been identified as the effectors of the type V system [25,26]. The type VI system employs higher eukaryotes and prokaryotes nucleotide-binding (HEPN) nuclease domain-containing effectors, including Cas13a, Cas13b, Cas13c and Cas13d [18].

Among all the CRISPR-Cas systems, the type I system accounts for 95% and is the most widely distributed. In many cases, heterologous proteins, such as Cas9 and Cpf1, are difficult to transform into bacteria and archaea due to their intrinsic toxicity, leading to a low genome editing efficiency. Thus, the type I CRISPR-Cas system was harnessed as an endogenous RNA-guided machinery for multiplex genome editing in prokaryotes [27–29]. The type II CRISPR-Cas9 system is the most popular genome editing tool and has been successfully applied in a broad range of organisms, such as bacteria, yeasts, plants, animals and human cells [30–33]. The type V CRISPR-Cpf1 system has emerged as an alternative to the CRISPR-Cas9 technology [34]. The genome editing activity of CRISPR-Cpf1 is not as robust as CRISPR-Cas9, but has higher targeting efficiency [35,36]. Given its powerful RNA recognition and cleavage ability, the type VI CRISPR-C2c2 system has been harnessed as a toolkit for RNA base editing, RNA knockdown, nucleic acid detection and transcript tracking [37]. Although lots of biochemical and structural studies have been reported concerning the composition and functional activities of these CRISPR-Cas systems, a comprehensive and systematic analysis of the diverse interference mechanisms of these genetic silencing systems is still lacking. In this review, we summarize the current knowledge related to these CRISPR-Cas effector complexes, which will deepen our understanding of the architecture of distinct types of CRISPR-Cas systems, and how crRNA-guided Cas proteins recognize and cleave invading nucleic acids. Furthermore, it will enhance the application of CRISPR-Cas systems as genome editing tools.

## THE TYPE I CRISPR-CAS SYSTEM: AN ENDOGENOUS TOOL FOR MULTIPLEX GENOME EDITING IN PROKARYOTES

The type I CRISPR-Cas complex is also named Cascade (CRISPR associated complex for antiviral defense), and is assembled by multiple Cas proteins and a crRNA [38,39]. The recognition of target DNA is initiated by PAM scanning, which assists in the unzipping of the base pairs adjacent to the PAM [40]. Then, the target DNA strand hybridizes with the crRNA spacer to form a heteroduplex, while the non-target DNA strand is displaced. This unique conformation is called an ‘R-loop’ [41]. After the formation of the Cascade/R-loop, Cas3 is recruited to degrade the target DNA [19,42]. Up to now, only the structures of type I-E and type I-F complexes have been determined, while the structures of the other type I DNA interference complexes remain unknown.

### Composition, structure and functional activities of the type I-E surveillance complex

**Figure 2.**
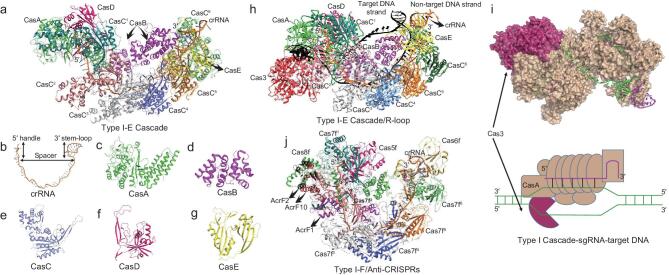
Structures of type I Cascade and its complexes. (a) Crystal structure of the RNA-guided type I-E CRISPR surveillance complex (PDB: 4U7U). The crRNA and five Cas proteins (CasA–E) are labeled. (b-g) Enlarged view of the 61-nt crRNA, as well as five Cas proteins (CasA–E). (h) Cryo-EM structure of the type I-E Cascade/R-loop/Cas3 from *Thermobifida fusca* (PDB: 6C66). Cas3 and the target dsDNA are colored red and black, respectively. The other subunits are colored the same as in Fig. 2a. (i) Cartoon showing the working model of the type I Cascade (PDB: 6U66). (j) Cryo-EM structure of the type I-F CRISPR surveillance complex bound with anti-CRISPRs AcrF1, AcrF2 and AcrF10 (PDB: 5UZ9 and 6B48). AcrF1, AcrF2 and AcrF10 are labeled and colored magenta, black and red, respectively.

The type I-E subtype is the most common and best-studied type I CRISPR-Cas system. It has been utilized as a programmable gene expression regulator, enabling the silencing of both heterologous and endogenous genes [43]. Furthermore, it was engineered to be a genetically encoded device, termed DNA interference (DNAi), which could sense transcriptional inputs and directly degrade user-defined DNAs [44]. The atomic structures of the *Escherichia coli* type I-E surveillance complex, and its complexes with ssDNA or dsDNA, were determined by X-ray diffraction [45,46]. The *E. coli* type I-E complex has a molecular weight of 405 kDa, comprising 11 subunits from five Cas proteins (CasA_1_, CasB_2_, CasC_6_, CasD_1_ and CasE_1_), as well as a 61-nt crRNA (Fig. 2a). The 61-nt crRNA is processed from pre-crRNA by CasE [47], which specifically recognizes and cuts the repeat sequences of pre-crRNA. The mature 61-nt crRNA is composed of an 8-nt 5′ handle, a 32-nt spacer sequence and a 21-nt 3′ stem-loop (Fig. 2b).

The overall structure of the type I-E complex adopts a seahorse shape containing 11 subunits, which are arranged into two layers (Fig. 2a). CasD, six copies of CasC, and CasE constitute the outer layer, while CasA and two copies of CasB form the inner layer (Fig. 2a). The outer and inner layers wrap each other to form a DNA-like double helix conformation. Within the outer layer, six copies of the CasC subunit display a symmetry-related helical alignment (Fig. 2a). CasD and CasE locate at the two ends, respectively (Fig. 2a). The CasA subunit is a two-domain protein, containing an N-terminal globular fold and a C-terminal four-helix bundle (Fig. 2c). The CasB subunit comprises two helix bundles connected by a loop (Fig. 2d). The structure of the CasC subunit resembles a right hand, consisting of a modified RRM domain (palm), a protruding β-hairpin (thumb) and a helical domain (fingers) (Fig. 2e). The CasD subunit also contains a modified RNA recognition motif (RRM) domain, with a β-hairpin protruding from the core (Fig. 2f). The CasE subunit contains two tandem RRM domains (Fig. 2g). Within the inner layer, CasA locates at one end, making extensive interactions with CasD. The CasB dimer sits in the groove enclosed by CasA, CasC and CasE (Fig. 2a). The 61-nt crRNA threads through the outer layer, connecting these subunits together. The 8-nt 5′ handle region of crRNA is sandwiched between CasA, CasD and the adjacent CasC^1^ (Fig. 2a). Six copies of the CasC subunit oligomerize along the spacer region of crRNA (Fig. 2a). After processing of the pre-crRNA, CasE remains tightly bound to the 3′ stem-loop of the crRNA (Fig. 2a).

The structure of the type I-E complex bound to target DNA reveals that the guide–target hybrid displays a ribbon-like conformation, in contrast to the double helix structure [48] (Fig. 2h). This is caused by kinks that occur in every sixth base pair in both strands of the hybrid. In addition to the Watson–Crick hydrogen bonding with the spacer region of the crRNA, the target DNA strand also interacts with the CasA, CasB dimer and the CasC^1^ subunits [48] (Fig. 2h). The type I-E complex adopts a promiscuous PAM recognition mode [49]. At least five PAM sequences, such as 5′-ATG, AGG, AAG, TAG and GAG, can initiate type I-E-mediated CRISPR interference [19,42]. More recently, Ke's group reported the cryogenic electron microscopy (Cryo-EM) structures of the type I-E Cascade/R-loop and type I-E Cascade/R-loop/Cas3 from *Thermobifida fusca* at atomic resolution [41,50]. The R-loop’s formation induces severe dsDNA bending at the PAM-proximal side, as well as a series of conformational changes in the type I-E Cascade [50]. Then, the type I-E Cascade/R-loop complex licenses Cas3 to bind (Fig. 2h-i). The recruitment of Cas3 mainly depends on the interactions between the Cas3 and CasA subunits in the Cascade complex in a fashion of conformation capture [50] (Fig. 2h-i). After Cas3-medited ssDNA nicking, the severed non-target strand DNA relocates to Cas3 helicase [50]. Finally, processive DNA degradation begins (Fig. 2i).

### Structures of the type I-F surveillance complex bound to anti-CRISPRs

Structural studies of the type I-F CRISPR-Cas system have benefited from the identification of phage-encoded anti-CRISPR proteins. Overall, 10 anti-CRISPR proteins targeting the type I-F genetic silencing machinery have been found [51,52]. To investigate the inhibition mechanisms of these anti-CRISPR proteins, Cryo-EM structures of the type I-F surveillance complex bound to the anti-CRISPR proteins AcrF1, AcrF2 and AcrF10, were determined [53–55]. The type I-F complex from *Pseudomonas aeruginosa* has a molecular weight of 350 kDa, comprising nine subunits from four Cas proteins (Cas5f_1_, Cas6f_1_, Cas7f_6_ and Cas8f_1_) as well as a 60-nt crRNA (Fig. 2j). The type I-F complex shows structural similarity to the previously described type I-E complex, with six copies of Cas7f as the backbone, one copy of Cas6f as the head and one copy of the Cas8f-Cas5f heterodimer as the tail (Fig. 2j). However, structural differences between the type I-E and type I-F complex still exist. The head and tail of the type I-F complex is positioned in close proximity, which causes a nearly closed ring architecture. In addition, the CasC^6^ subunit of the type I-E complex rotates 180 degrees to form a region for binding to dsDNA, which is not observed in the corresponding subunit of the type I-F complex.

The overall structure of the full-length crRNA in the type I-F complex resembles a string that tethers distinct protein subunits together. Extensive intermolecular interactions are formed between the crRNA and the protein subunits. The crRNA recognition modes between type I-F and I-E complexes are tremendously similar. Briefly, the 5′ handle region of the crRNA is sandwiched between the Cas5f, Cas8f and adjacent Cas7f^6^ subunits (Fig. 2j). The backbone region of the crRNA threads through the multiple copies of Cas7f (Fig. 2j). The 3′ stem-loop is recognized by the Cas6f subunit (Fig. 2j). As observed in these complex structures, all of these anti-CRISPR proteins (AcrF1, AcrF2 and AcrF10) locate in positions that partially overlap with the binding sites of target DNAs (Fig. 2j). These anti-CRISPR proteins adopt a similar inhibition strategy by interfering with the type I-F silencing machinery to recognize the target DNAs.

## TYPE II CRISPR-CAS9: A HIGHLY EFFICIENT GENOME EDITING TOOL IMPLEMENTED IN A BROAD RANGE OF ORGANISMS

Cas9 is the best-characterized member of the class II CRISPR-Cas system, which has been widely used as a tool for genome engineering and gene expression control [56,57]. Interestingly, the CRISPR-Cas9 gene locus encodes another noncoding RNA, named tracrRNA [58]. The sequence of the tracrRNA is partially complementary to the repeat segment of the crRNA, forming a tracrRNA–crRNA duplex. Cas9 is activated through its assembly with this tracrRNA–crRNA duplex to form a Cas9-crRNA-tracrRNA surveillance complex (Table 1) [58]. The tracrRNA–crRNA duplex can be engineered to a chimeric structure by connecting the 5′-end of the tracrRNA to the 3′-end of the crRNA, named the single-guide sgRNA). The Cas9-sgRNA two-component system simplifies the applications of CRISPR-Cas9 technology in genome editing. The accurate selection of target DNA depends on a PAM sequence, as well as the base pairing between the target DNA strands with the ‘seed’ sequence within the guide segment of the crRNA [58]. Cas9 proteins are widespread among the bacterial kingdom, differing in both sequence and size. The Cas9 protein found in *Streptococcus pyogenes* (SpCas9) is the most common and widely studied one.

**Table 1.** Comparison of distinct types of CRISPR-Cas effectors.

	Type I-E Cascade	Type II Cas9	Type V-A Cfp1	Type V-B C2c1	Type VI C2c2
Protein composition	Multiple subunits	Single subunit	Single subunit	Single subunit	Single subunit
Pre-crRNA processing	Mediated by accessory protein	Mediated by accessory protein	Self-processing	Mediated by accessory protein	Self-processing
RNA composition	crRNA	tracrRNA/crRNA	crRNA	tracrRNA/crRNA	crRNA
Substrate	dsDNA	dsDNA	dsDNA	dsDNA	ssRNA
PAM	Promiscuous PAMs	G-rich	T-rich	T-rich	Non-G PFS
PAM recognition pattern	Both DNA strands	NT strand	Both DNA strands	Both DNA strands	T strand
Length of guide–target duplex	32	20	20	20	24
Catalytic domain	HD (Cas3)	HNH and RuvC	RuvC-Nuc	RuvC-Nuc	2*HEPN

NT: non-target; T: target.

### Domain organization, structure and functional activities of the type II CRISPR-Cas9 system

**Figure 3.**
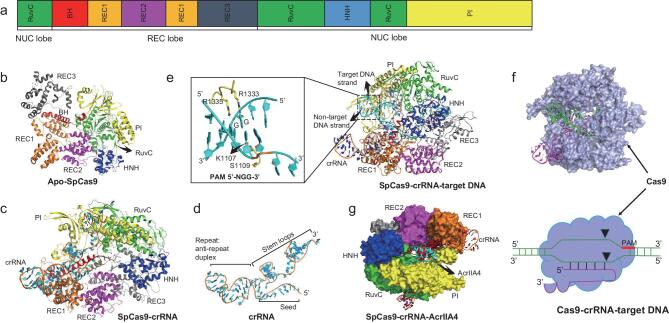
Domain organization and structures of Cas9 and its complexes. (a) Domain organization of Cas9. (b) Crystal structure of Apo-SpCas9 (PDB: 4CMP). (c) Crystal structure of SpCas9 in complex with sgRNA (PDB: 4ZT0). (d) Cartoon showing the sgRNA observed in the structure of the SpCas9-sgRNA binary complex. (e) Crystal structure of SpCas9 in complex with sgRNA and target DNA (5′-NGG-3′ PAM) (PDB: 4UN3). (f) Cartoon showing the working model of type II-A SpCas9 (PDB: 4UN3). (g) The complex structure of SpCas9-sgRNA-AcrIIA4 (PDB: 5XBL). sgRNA and AcrIIA4 are colored orange and cyan, respectively. In panels (b, c, e and f), SpCas9 domains are colored the same as panel (a).

Over the past a few years, several structural studies on SpCas9 have been reported, including the structures of apo-form SpCas9, the SpCas9-sgRNA binary complex and the SpCas9-sgRNA-target DNA ternary complex [59–62]. SpCas9 adopts a bi-lobed architecture, comprising a recognition (REC) lobe and a nuclease (NUC) lobe (Fig. 3a). The REC lobe is composed of a bridge helix motif (BH), a REC1 domain (Helical-I), a REC2 (Helical-II) domain and a REC3 (Helical-III) domain (Fig. 3a-b). The NUC lobe consists of a RuvC domain, a HNH domain, and a PAM-interacting (PI) domain (Fig. 3a-b). The REC domain is composed of multiple helix bundles, showing no structural similarity to any known proteins. Upon sgRNA loading, the REC lobe undergoes substantial conformational changes, inducing the formation of a central channel to accommodate the guide RNA–target DNA heteroduplex [60]. Target DNA binding also causes a series of conformational changes of SpCas9. The HNH catalytic domain moves toward the target DNA strand and a modest shift is observed in the REC lobe [60]. This substrate-induced fit mechanism ensures the optimal positioning of target DNA for cleavage.

In the SpCas9-sgRNA binary complex, the sgRNA displays an L-shape, comprising a crRNA and a tracrRNA connected by an artificial tetraloop (Fig. 3c-d). The crRNA is composed of guide and repeat segments (Fig. 3d). The tracrRNA consists of an antirepeat segment and three stem-loops (Fig. 3d). The repeat segment of crRNA and the antirepeat segment of tracrRNA form the repeat–antirepeat duplex (Fig. 3d). In the absence of target DNA, only the 10-nt seed sequence in the guide segment of crRNA is observed, which adopts an A-form conformation (Fig. 3c-d). In the SpCas9-sgRNA-target DNA ternary complex, a full 20-nt guide sequence is present, which hybridized with the target DNA strand to form the guide–target duplex (Fig. 3e). The guide–target and repeat–antirepeat duplexes, as well as the DNA duplex containing the PAM sequence, locate in the channel formed by the REC and NUC lobes (Fig. 3e). The stem-loops of tracrRNA are solvent exposed, making extensive interactions with the REC1, RuvC and PI domains.

After target DNA unzipping, one of the DNA strands (the target strand) hybridizes with the guide region of the crRNA to form the crRNA–DNA heteroduplex, whereas the other one (the non-target DNA) is displaced. This represents a transient and pre-cleaved state, named the R-loop conformation [63]. The formation of the R-loop structure plays an important role for placing each DNA strand for catalysis. Finally, Cas9 cleaves the target and non-target DNA strands using the HNH and RuvC nuclease domains, respectively, making a blunt double-stranded break (Fig. 3f). A near-atomic Cryo-EM structure of the SpCas9-R-loop complex clearly shows that the displaced non-target DNA strand protrudes into the active site of the RuvC domain [63]. Another 5.2 Å Cryo-EM structure of SpCas9-sgRNA-target DNA captures a conformation of SpCas9 in which the HNH domain is close to the target DNA cleavage site [64]. These Cryo-EM structures strongly support the present understanding of the catalytic mechanism of the type II CRISPR-Cas9 system. Besides SpCas9, lots of crystal structures of Cas9 homologs were determined, including Cas9 from *Actinomyces naeslundii*, *Campylobacter jejuni*, *Francisella novicida* and *Staphylococcus aureus* [62,65,66]. These Cas9 homologs share similar domain composition and structural features. Though distinct sequence preferences for PAMs and crRNA–tracrRNA scaffolds exist among these proteins, the mechanisms for substrate binding, PAM selection, target DNA unzipping and substrate cleavage are quite similar.

### Structure of SpCas9 variants with broad PAM compatibility and enhanced specificity

SpCas9 specifically recognizes the 5′-NGG-3′ PAM sequence through the PI domain. Two conserved residues (Arg^1333^ and Arg^1335^) in the PI domain are inserted into the major groove of the target DNA duplex, forming hydrogen bonds with the two guanine bases in the PAM (Fig. 3e). Another two residues (Lys^1107^ and Ser^1109^) in the same domain serve as a phosphate lock, recognize the phosphate group immediately upstream of the PAM and making a kink in the target DNA strand (Fig. 3e). Thus, Watson–Crick base pairs close to the PAM are separated. PAM recognition plays a key role in preventing the CRISPR-Cas9 immune system from targeting the host's own genetic material and facilitates the unzipping of the PAM adjacent target DNA duplex. However, the specific PAM recognition pattern limits the applications of the Cas9-mediated genome editing tool. To break this barrier, three SpCas9 variants were screened by utilizing a method named bacterial selection-based directed evolution, which could recognize the 5′-NGAN-3′, 5′-NGNG-3′ and 5′-NGCG-3′ PAMs [67]. Structures of these Cas9 variants in complex with sgRNA and target DNAs containing non-canonical PAMs revealed that structural rearrangement occurs in the PAM region of target DNA, which allows the SpCas9 variants to form compact interactions with the altered PAM nucleotides through an induced fit mechanism. More recently, Liu's group screened a Cas9 variant (xCas9) through phage-assisted continuous evolution [68]. The xCas9 possesses the broadest PAM compatibility among Cas9 family proteins and has high DNA specificity. Besides xCas9, several other SpCas9 variants with high fidelity and enhanced specificity have also been reported, including SpCas9-HF1, HypaCas9 and eSpCas9 [69–71]. These SpCas9 variants will improve the application of CRISPR-Cas9 technology in the future by reducing off-target cleavage and enhancing precision genome editing.

### Structure of SpCas9 in complex with anti-CRISPR

Although CRISPR-Cas9 is the most powerful genome editing tool so far and has been successfully applied in a broad range of organisms [31,33], the high ratio of off-target effects of CRISPR-Cas9 technology cannot be ignored [72]. AcrIIA4 is an already known anti-CRISPR protein that is encoded by *Listeria monocytogenes* prophage, which has been reported to completely inhibit the activity of SpCas9 [73]. AcrIIA4 adopts a ‘triangle’ fold, comprising three antiparallel β-strands with three α-helices at one side (Fig. 3g). The structure of AcrIIA4 in complex with the sgRNA-loaded SpCas9 reveals that AcrIIA4 interacts with the REC, PI, and RuvC domains of SpCas9, sterically blocking the PAM binding site [74] (Fig. 3g). These studies have facilitated the application of AcrIIA4 as an ‘off-switch’ tool to control the activity of SpCas9.

## TYPE V CRISPR-CPF1: AN ALTERNATIVE GENOME EDITING TOOL WITH HIGHER TARGETING EFFICIENCY

According to its distinct effector proteins, the type V CRISPR-Cas system can be divided into three subtypes, including Cas12a-Cpf1 (V-A), Cas12b-C2c1 (V-B) and Cas12c-C2c3 (V-C). Cpf1 was first identified in 2015 and specifically cleaves both strands of the target DNA [26]. Similar to Cas9, Cpf1 is a single RNA-guided endonuclease, showing robust genome editing activity in human cells [75]. However, CRISPR-Cpf1-mediated DNAi possesses four unique features. First, Cpf1 processes the pre-crRNA utilizing divalent cation-independent endonuclease activity, and the mature crRNA does not require an additional *trans*-activating crRNA (tracrRNA) (Table 1) [76]. Second, Cpf1 recognizes T-rich PAMs and PAM-complementary nucleotides, whereas Cas9 recognizes G-rich PAMs (Table 1) [77]. Third, Cpf1 cleaves both strands of the target dsDNA with a staggered cut (4- or 5-nt 5′ overhang), in contrast to the blunt ends produced by Cas9 [78]. Fourth, Cpf1 contains only a detectable endonuclease domain, RuvC, whereas Cas9 possesses another HNH endonuclease domain (Table 1) [78,79]. Similar to Cpf1, C2c1 also recognizes the T-rich PAMs (Table 1) [25]. However, C2c1-mediated DNA cleavage requires both the crRNA and tracrRNA for activity (Table 1), and generates a staggered double-stranded break with a 7-nt 5′ overhang [25]. The cleavage activity of C2c1 is temperature-dependent, with 40–60°C as the optimal cleavage temperature. This feature limits the utilization of C2c1 for genome editing application. C2c3 was reported with C2c1 at the same time, due to containing RuvC-like endonuclease domains [25]. However, the detailed domain composition, structure and activity of C2c3 remain to be further investigated.

### Domain organization and structure of CRISPR-Cpf1

**Figure 4.**
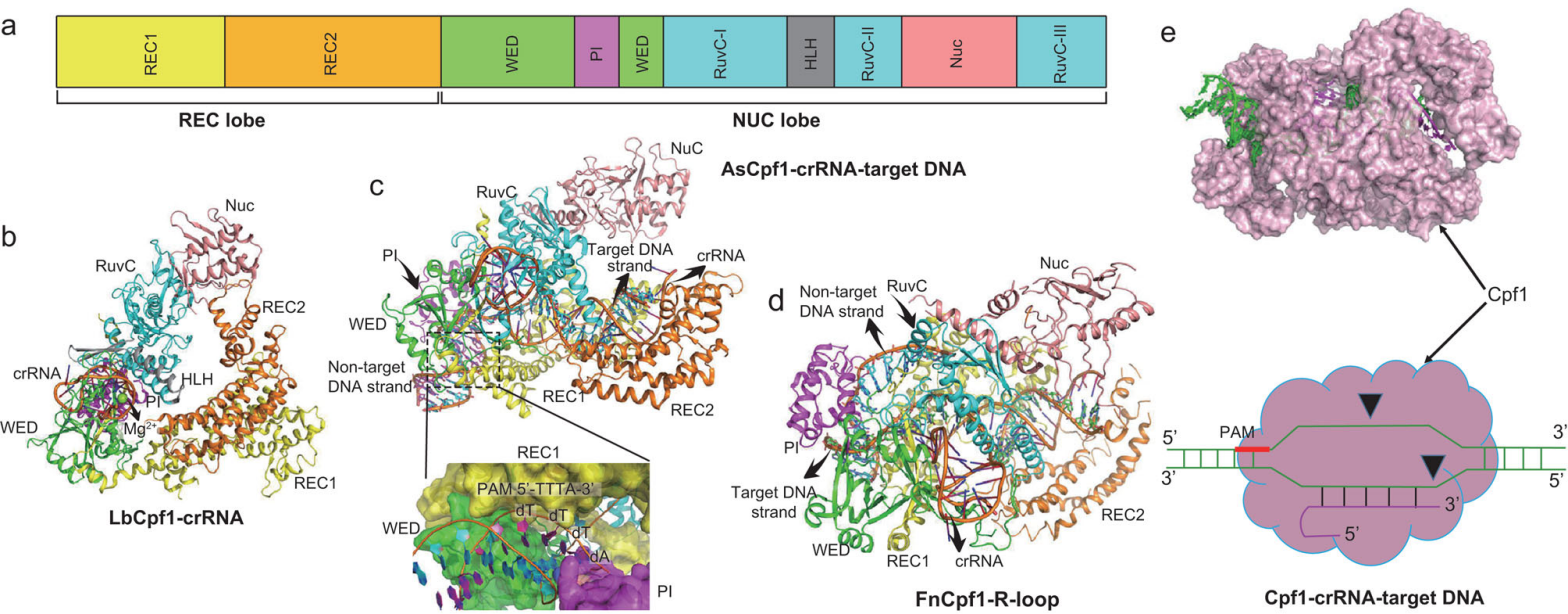
Domain organization and structures of Cpf1, and its complex with nucleic acids. (a) Domain organization of Cpf1. (b) Crystal structure of LbCpf1-crRNA binary complex (PDB: 5ID6). (c) Crystal structure of AsCpf1 in complex with crRNA and target DNA (5′-TTTA-3′ PAM) (PDB: 5B43). (d) Crystal structure of FnCpf1-R-loop complex (PDB: 5MGA). In panels (b–d), Cpf1 domains are colored the same as panel (a). (e) Cartoon shows the working model of type V-A Cpf1 (PDB: 5B43).

The crystal structure of *Lachnospiraceae* bacterium ND2006 Cpf1 (LbCpf1) in complex with a 43-nt crRNA was first determined at a resolution of 2.38 Å [80]. Similar to the type II Cas9 effector, LbCpf1 displays a bi-lobed architecture, consisting of a REC lobe and a NUC lobe (Fig. 4a). The REC lobe is composed of two helical domains, named Helical-I (REC1) and Helical-II (REC2) (Fig. 4a). The NUC lobe consists of an oligonucleotide-binding domain (OBD or WED), a looped-out helical domain (LHD or PI), a HLH domain (BH), a Nu domain and a RuvC domain (Fig. 4a). These domains enclose a triangle-like shape, with a large cavity at the center where the crRNA and target dsDNA are placed (Fig. 4b-d). In the complex structure of LbCpf1-crRNA, only the repeat sequence of the crRNA is well defined regarding its electron density, whereas the guide sequence is not observed (Fig. 4b). As shown in the complex structure, the repeat region of crRNA is highly distorted, adopting a stem-loop-like conformation (Fig. 4b). It makes extensive intermolecular interactions with the WED and RuvC domains of LbCpf1 (Fig. 4b). It is worth noting that an Mg(H_2_O)_6_^2+^ ion is observed in the center of the repeat region of crRNA, which functions to stabilize its unique conformation (Fig. 4b).

### Target DNA recognition and functional activity of CRISPR-Cpf1

Soon after the report of the complex structure of LbCpf1-crRNA, Nureki's group determined the crystal structure of *Acidaminococcus sp*. Cpf1 (AsCpf1) complexed with the crRNA and target DNA containing the 5′-TTTN-3′ PAM at a resolution of 2.8 Å [77]. In this complex structure, it can clearly be seen that the crRNA–target DNA heteroduplex lies in the central cavity enclosed by the REC1, REC2, WED and RuvC domains (Fig. 4c). The crRNA is composed of a 19-nt 5′-handle and a 24-nt guide segment. The PAM sequence is recognized by the REC1, WED and PI domains through both the base and shape readout mechanisms [77] (Fig. 4c). Combining the structural and biochemical information, the authors proposed that the Nuc domain is also an endonuclease domain, although it shows no structural and sequence similarity to any identified nucleases. Subsequently, the crystal structure of the *F*. *novicida* Cpf1 (FnCpf1)-R-loop complex was determined, which perfected our understanding of the process of recognition, unzipping and cleavage of the target DNA [81,82] (Fig. 4d). Putting all of these structures together, a working model for Cpf1 was proposed. Firstly, the Cpf1-crRNA complex undergoes a conformational change upon target DNA binding to allow PAM scanning. The recognition of PAM induces the HLH domain to adopt a ‘flap-on’

conformation and the LKL helix to insert into the double-stranded DNA [81]. Then, the base pairs adjacent to the PAM are unzipped, which allows the hybridization of the target DNA strand with the crRNA. Finally, cleavage occurs on both strands of the target DNA to generate an overhang (Fig. 4e). In LbCpf1 and AsCpf1, mutations of the catalytic residues in the Nuc domain impact the cleavage of the target DNA strand, whereas mutations of the catalytic residues in the RuvC domain disturb the cleavage of both strands of the target DNA. Extensive mutational analysis of the putative active residues in FnCpf1 supports the idea that a single active site located at the interface of the Nuc and RuvC domains takes charge of cleaving both of the target and non-target DNA strands.

### Structural plasticity of PAM recognition by CRISPR-Cpf1

Besides the optimal canonical 5′-TTTN-3′ PAM, Cpf1 recognizes the suboptimal non-canonical PAMs, including 5′-TCTA-3′, 5′-TCCA-3′ and 5′-CCCA-3′ [83]. However, LbCpf1 recognizes the canonical PAM more efficiently than these non-canonical PAMs. Structures of LbCpf1 complexed with these non-canonical PAMs containing DNAs were determined [83]. Both of the canonical and non-canonical PAM duplexes are located in a channel formed by the REC1, WED and PI domains. Structural superposition of these four structures indicated that the PI domain moves outward in these non-canonical PAM-containing structures, which enlarged the non-canonical PAM binding channel [83]. The structural plasticity of the PAM binding channel renders Cpf1 able to recognize both of the canonical and non-canonical PAMs.

### Domain organization, structure and functional activities of CRISPR-C2c1

**Figure 5.**
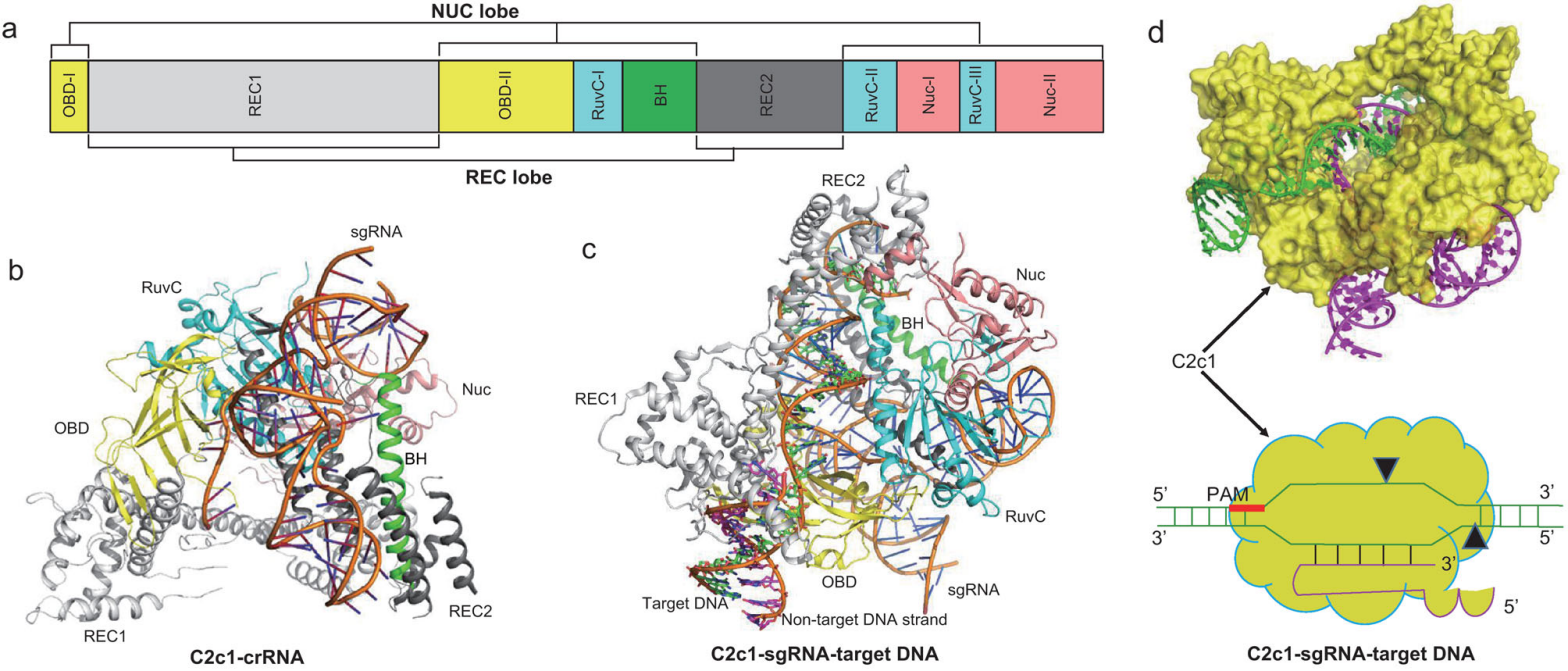
Domain organization and structures of C2c1 and its complex with nucleic acids. (a) Domain organization of C2c1. (b) Crystal structure of AacC2c1-sgRNA binary complex (PDB: 5U34). (c) Crystal structure of AacC2c1 in complex with crRNA and target DNA (5′-TTC-3′ PAM) (PDB: 5B43). In panels (b and c), C2c1 domains are colored the same as panel (a). (d) Cartoon shows the working model of type V-B C2c1 (PDB: 5B43).

The structures of the C2c1-sgRNA binary complex and the C2c1-sgRNA-target DNA ternary complex were also determined [84–86]. Similar to Cas9 and Cpf1, C2c1 contains two lobes, displaying an overall ‘crab claw’ fold. The domain organization of C2c1 resembles that of Cpf1, except that it lacks the PI domain (Fig. 5a). In addition, the REC2 domain is in close proximity to the REC1 domain in Cpf1, whereas it is inserted between the BH and RuvC-II motifs in C2c1 (Fig. 5a). The Nuc domain of C2c1 is divided into two parts by the RuvC-III motif and shows low structural similarity to that in Cpf1 (Fig. 5a). The sgRNA observed in the complex structure is a chimeric tracrRNA–crRNA duplex, which is engineered by connecting the 5′-end of the crRNA to the 3′-end of tracrRNA (Fig. 5b). The sgRNA makes extensive interactions with the OBD, REC, RuvC and Nuc domains of C2c1 (Fig. 5b). The guide region of crRNA hybridizes with the target DNA strand, located in a channel enclosed by the REC, BH, OBD and RuvC domains, whereas the tracrRNA is solvent exposed (Fig. 5c). In contrast to the relaxed PAM recognition pattern of Cas9 and Cpf1, C2c1 recognizes the PAM with stringent specificity [84]. In addition, the cleavage site on the target strand locates within the guide–target duplex for Cas9 and Cpf1, whereas C2c1 cleaves the target strand at a site outside the guide–target duplex [84] (Fig. 5d). Interestingly, the structure of C2c1-crRNA-extended target DNA reveals that both the target and non-target strand extensions are inserted into the same RuvC catalytic pocket [85]. This provides evidence that type V-Cas12 effectors may cleave both the target and non-target DNA strands using a single active site. However, the precise catalytic mechanism of how the RuvC active site of Cas12 nucleases cleaves both the target and non-target DNA strands independently needs further investigation.

## TYPE VI CRISPR-CAS13: A TOOL FOR RNA EDITING AND RAPID NUCLEIC ACID DETECTION

The type VI CRISPR-Cas system is solely dedicated to RNA-guided RNA-targeting of the adaptive immune system, and is characterized by the single HEPN domain-containing effector Cas13. In addition to being a tool for RNA base editing, Cas13 has been developed as a platform for rapid nucleic acid detection, named SHERLOCK [87]. Cas13 possesses two RNase activities, which are mechanistically distinct from each other. First, it can cleave and process the pre-crRNA to generate mature crRNA. Second, it can recognize and degrade target RNA under the guidance of crRNA. Upon target RNA binding and activation, Cas13 also possesses the ability to cleave unrelated RNA molecules without any complementarity to the guide region of crRNA. Up to now, four Cas13 family proteins have been identified, including Cas13a, Cas13b, Cas13c and Cas13d. Among these proteins, Cas13a (C2c2) is the one that was first identified and is the best studied.

### Domain organization and structure of CRISPR-C2c2

**Figure 6.**
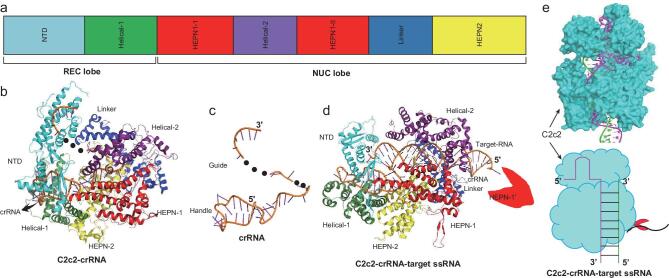
Domain organization and structures of C2c2 and its complex with nucleic acids. (a) Domain organization of C2c2. (b) Crystal structure of LshC2c2-crRNA binary complex (PDB: 5WTK). (c) Cartoon shows the crRNA observed in the structure of the C2c2-crRNA binary complex. (d) Crystal structure of LbuC2c2-crRNA-target RNA ternary complex (PDB: 5XWP). In panels (b and d), C2c2 domains are colored the same as panel (a). (e) Cartoon shows the working model of type VI-A C2c2 (PDB: 5XWP).

To understand the mechanism of per-crRNA processing and crRNA-guided ssRNA degradation, the structures of apo-C2c2, C2c2-crRNA and C2c2-crRNA-ssRNA were determined. In 2016, Wang's group determined the crystal structures of *Leptotrichia shahii* C2c2 (LshC2c2) and its complex with a crRNA at a resolution of 2.65 Å and 3.5 Å, respectively. [88]. Similar to other class II effectors, the structure of LshC2c2 also displays a bi-lobed architecture, consisting of a REC lobe and a NUC lobe (Fig. 6a-b). The REC lobe is composed of an N-terminal domain (NTD) and a Helical-1 domain (Fig. 6a). The NUC lobe consists of two HEPN domains, a Helical-2 domain and a linker domain (Fig. 6a). The crRNA is composed of a 5′-handle region and a guide segment, located in a groove enclosed by the REC lobe and the NUC lobe (Fig. 6b-c). The REC lobe mainly recognizes the 5′-handle region, whereas the NUC lobe interacts with the guide segment (Fig. 6b). LshC2c2 recognizes the 5′-handle in a sequence-specific manner. Both the structure and sequence of the 5′-handle region are vital for the dual RNase activities of LshC2c2[88]. The crRNA guide segment in the structure of LshC2c2-crRNA is incomplete. The 5′-end of the guide adopts a U-shape, embedded deeply in a hole enclosed by the HEPN2 domain and the linker domain (Fig. 6b). The central part of the guide is unstructured and missing, indicating that it may be flexible and exposed to the bulk solvent (Fig. 6b). The 3′-end of the guide sits at the concave surface of the NTD domain (Fig. 6b). The central part and 3′-end of the guide works as a seed sequence to hybridize with the target RNAs [89]. Upon crRNA binding, the Helical-2 domain of LshC2c2 undergoes a large conformational change, moving towards the linker and HEPN2 domains to enclose a crRNA binding groove [88].

### Target RNA recognition and functional activity of CRIPSR-C2c2

Subsequently, Wang's group reported the crystal structure of *L*. *buccalis* C2c2 (LbuC2c2) in complex with a crRNA and a target RNA, as well as a Cryo-EM structure of the LbuC2c2-crRNA complex at a resolution of 3.08 Å and 3.2 Å [90], in which nearly all nucleotides of the crRNA are observed (Fig. 6d). The target RNA forms 28 base pairs with the guide region of crRNA in the structure of LbuC2c2-crRNA-target RNA, which leaves two nucleotides (one at the 5′-end and the other one at the 3′-end) flipping out of the target–guide RNA duplex (Fig. 6d). The nucleotide at the 5′-end of the target RNA inserts into the catalytic pocket of the HEPN1 domain of a neighboring LbuC2c2 molecule (Fig. 6d). The 3′-end nucleotide locates in a groove enclosed by the NTD domain and the Helical-1 domain (Fig. 6d). In addition to base pairing with the guide region of crRNA, the target RNA also interacts with the HEPN1, Helical-2, and linker domains of LbuC2c2 (Fig. 6d). Upon target RNA binding, significant conformational rearrangements occur in both LbuC2c2 and the crRNA, which makes a suitable binding groove for the crRNA–target RNA duplex [90]. Meanwhile, the guide region of the crRNA changes its conformation from multiple turns to a regular A-form helix [90].

C2c2 has two separate catalytic centers for its dual RNase activities. The Helical-1 and HEPN2 domains are found to be responsible for pre-crRNA processing in LshC2c2 and LbuC2c2, respectively [90], While the HEPN1 domain together with the HEPN2 domain plays the key role in target and collateral degradation [90]. The formation of the guide–target RNA duplex causes the HEPN1 domain to move toward the HEPN2 domain. Two catalytic residues on each HEPN domain are brought together to create a composite HEPN catalytic center. The activated C2c2 can cleave any exposed ssRNAs, including the target RNA extending from the guide–target complementary region and the free RNAs in solution (Fig. 6e).

## CONNECTION BETWEEN THE STRUCTURES AND POTENTIAL GENOME EDITING USAGE

In biology, an important insight is that structure determines the function. Studying structural information can enable us to better understand the functional activities of CRISPR-Cas systems and promote the application of genome editing. Based on the 3D structures, Cas9 and Cpf1 variants with altered PAM specificity have been designed. These variants have enabled the editing of gene sites that were are targeted by wild-type (wt) Cas9 or Cpf1 in human cells. Structural information can also guide sgRNA design. For Cas9, truncated sgRNAs with 17- or 18-nt guide sequences have shown much reduced off-target activity in human cells without reducing on-target genome editing efficiency [91]. Another strategy that can reduce the off-target activity of Cas9 is the mutation of amino acid residues in charge of stabilizing the R-loop structure. In accordance with this principle, four Cas9 variants with high fidelity and enhanced specificity have been designed, including eSpCas9, SpCas9-HF, HypaCas9 and evoCas9 [69,70,92]. In particular, evoCas9 has shown 79-fold higher fidelity than wtCas9 [92]. As mentioned above, Cas9 possesses two catalytic domains, HNH and RuvC. Inactivation of one of the catalytic residues generates a Cas9 variant (nickase) that can only cleave either the target DNA or non-target DNA strand. Cas9 nickases show reduced off-target activity and facilitated overhang-based cloning [93,94]. More recently, structure-based inactivation of Cas9 (dCas9), Cpf1 (dCpf1) and C2c2 (dC2c2) proteins, which are fused to specific effector proteins for base-specific genome editing [95], have been widely used.

## CONCLUDING REMARKS

The CRISPR-Cas adaptive immune systems found in prokaryotes are thought to be one of the most significant discoveries in life science. Given the powerful applications in healthcare and agriculture, CRISPR-Cas systems have attracted much attention in recent years as a genome engineering tool. The recent elucidation of the biochemical mechanisms involved, as well as structural studies of several Cas proteins and their complexes with nucleic acids, have increased our understanding of the CRISPR-Cas genetic silencing machinery. In this review, we have focused on recent advances in structural studies of these CRISPR-Cas-mediated genome editing tools. The architecture of the type I Cascade complex shows similarities with the type III Csm/Cmr complex, supporting the hypothesis that these two types of CRISPR immune systems may have evolved from a common ancestor [96], particularly as both of them contain a crRNA-binding platform composed of multiple copies of Cas7 family proteins. The class II CRISPR-Cas effectors, such as type II Cas9, type V Cas12 and type VI Cas13, share low sequence similarity and adopt distinct domain organizations. A phylogenetic study has suggested that these types of effectors may evolve independently from distinct members of the TnpB family nucleases [25]. The class II CRISPR-Cas effectors recognize target nucleic acids dependent on the PAM sequence or 3′-PFS (protospacer-flank site). Cas9 and Cas12 recognize the PAMs in a sequence-specific manner, whereas Cas13 interacts with the 3′-PFS non-specifically (Table 1). In contrast to Cas9, which interacts only with the PAM in the non-target strand, Cas12 recognizes double DNA strands at the PAM region (Table 1). Cas13 cleaves both target and collateral RNAs in a non-specific manner (Table 1). Conversely, Cas9 and Cas12 cleave target DNA or RNA at specific sites (Table 1).

In the past decade, extensive research has built a framework for our understanding of the composition, structure and functional activities of distinct types of CRISPR-Cas systems. However, how CRISPR-based technology can be applied to achieve efficient and precise genome engineering still needs further exploration. We proposed that scientists need to devote more research effort in at least two research fields. First is the identification of novel proteins or small molecules that regulate the function of CRISPR-Cas machinery, and understanding their mechanism of action. Over the past a few years, scientists have found that viruses and mobile genetic elements encode a type of proteins, named anti-CRISPR, which can destroy the highly prevalent CRISPR-Cas immune systems of prokaryotes. Lots of anti-CRISPR proteins targeting the type I Cascade complex and type II Cas9 have been identified [97–99]. These proteins are diverse in terms of their sequence and structure, inhibiting target CRISPR-Cas effectors with different strategies. In 2017, Huang's group determined the first structure of the class II anti-CRISPR protein AcrIIA4 in complex with SpCas9 and an sgRNA, which has provided a structural basis for the development of tools to eliminate the genome-wide off-target activity of SpCas9 [74]. In contrast, to repress the activity of CRISPR-Cas effectors, some other accessory proteins, such as Csx28 and WYL1, have been found that could enhance Cas13-mediated RNA interference [100,101]. More recently, Zhu's group identified two small molecules (VE-822 and AZD-7762) through an unbiased drug selection-based platform [102]. These two compounds can stimulate CRISPR-Cpf1-mediated precise genome editing. Second is structure-based engineering or continuous directed evolution of CRISPR-Cas effectors to improve their application in genome editing, transcriptional activation or clinical viral nucleic acid detection. Many scientists has succeeded in engineering Cas9 and Cpf1 with altered PAM specificities to increase the genome targeting range [68,103–105]. These studies will boost the use of CRISPR-Cas9/Cpf1 systems in genome editing applications.

A close connection between the structural studies and potential genome editing usage of the CRISPR-Cas effector proteins exists. Learning structural information enables us to understand the domain architecture and conformational activation of these effectors, and improves their application for genome editing. Many strategies have been employed to overcome the limitations of off-target effects and stringent requirements for the PAM sequence. Structure-based engineering of the amino acid residues neighboring the PAM binding region yields lots of Cas9 and Cpf1 variants with expanded targeting spaces. A strategy that introduces non-base-specific interactions to compensate base-specific interaction is applied. In accordance with this principle, a SpCas9 variant SpCas9-NG was designed, which recognized relaxed NG PAMs [106]. In addition, structure-based engineering of the amino acid residues in charge of stabilizing the R-loop’s structure led to the discovery of evoCas9, which displayed 79-fold higher fidelity than wtCas9 [92]. More recently, Cas9- and Cas12-directed DNA base editors, and a Cas13-directed RNA base editor, have been developed, utilizing catalytically inactivated CRISPR-Cas effector proteins together with other enzymes [95]. Taken together, humans are gradually mastering the ability to efficiently and precisely edit the genomes of cells.
